# Microbiome Composition and Dynamics of a Reductive/Oxidative Bioelectrochemical System for Perchloroethylene Removal: Effect of the Feeding Composition

**DOI:** 10.3389/fmicb.2022.951911

**Published:** 2022-07-18

**Authors:** Maria L. Di Franca, Bruna Matturro, Simona Crognale, Marco Zeppilli, Edoardo Dell’Armi, Mauro Majone, Marco Petrangeli Papini, Simona Rossetti

**Affiliations:** ^1^Water Research Institute-National Research Council (IRSA-CNR), Rome, Italy; ^2^Department of Chemistry, Sapienza University of Rome, Rome, Italy

**Keywords:** reductive dechlorination, oxidative dechlorination, bioelectroremediation, chlorinated ethylenes, groundwater remediation, PCE

## Abstract

Chlorinated solvents still represent an environmental concern that requires sustainable and innovative bioremediation strategies. This study describes the microbiome composition of a novel bioelectrochemical system (BES) based on sequential reductive/oxidative dechlorination for complete perchloroethylene (PCE) removal occurring in two separate but sequential chambers. The BES has been tested under various feeding compositions [i.e., anaerobic mineral medium (MM), synthetic groundwater (SG), and real groundwater (RG)] differing in presence of sulfate, nitrate, and iron (III). In addition, the main biomarkers of the dechlorination process have been monitored in the system under various conditions. Among them, *Dehalococcoides mccartyi 16S rRNA* and reductive dehalogenase genes (*tceA*, *bvcA*, and *vcrA*) involved in anaerobic dechlorination have been quantified. The *etnE* and *etnC* genes involved in aerobic dechlorination have also been quantified. The feeding composition affected the microbiome, in particular when the BES was fed with RG. *Sulfuricurvum*, enriched in the reductive compartment, operated with MM and SG, suggesting complex interactions in the sulfur cycle mostly including sulfur oxidation occurring at the anodic counter electrode (MM) or coupled to nitrate reduction (SG). Moreover, the known *Mycobacterium* responsible for natural attenuation of VC by aerobic degradation was found abundant in the oxidative compartment fed with RG, which was in line with the high VC removal observed (92 ± 2%). *D. mccartyi* was observed in all the tested conditions ranging from 8.78E + 06 (with RG) to 2.35E + 07 (with MM) 16S rRNA gene copies/L. *tceA* was found as the most abundant reductive dehalogenase gene in all the conditions explored (up to 2.46 E + 07 gene copies/L in MM). The microbiome dynamics and the occurrence of biomarkers of dechlorination, along with the kinetic performance of the system under various feeding conditions, suggested promising implications for the scale-up of the BES, which couples reductive with oxidative dechlorination to ensure the complete removal of highly chlorinated ethylene and mobile low-chlorinated by-products.

## Introduction

Chlorinated aliphatic compounds (CAHs) are widespread contaminants that pose serious environmental and health concerns. Due to extensive applications, careless handling and storage, spills, and leakages, CAHs are among the most abundant classes of groundwater contaminants (Hatt and Löffler, 2012). Chlorinated solvents, including perchloroethylene (PCE), migrate downward to form dense non-aqueous phase liquids (DNAPLs), which constitute a source of contamination that may last for decades in groundwater (McCarty et al., 2007). Remediation technologies conventionally consist of separation techniques (i.e., ion exchange, reverse osmosis, or nanofiltration) (Altalyan et al., 2016) or permeable reactive barriers (Obiri-Nyarko et al., 2014). Since the discovery of the biological reductive dechlorination (RD) process, many bioengineering solutions have been successfully applied for *in situ* CAH removal. RD is an anaerobic process that allows bacterial growth with CAHs’ biodegradation up to the harmless ethylene in the presence of electron donors (i.e., hydrogen or fermentable organic substrates) (Hug and Edwards, 2013; Paul et al., 2016). RD occurs through a step-by-step process where highly chlorinated compounds, including PCE and trichloroethylene (TCE), are sequentially reduced to *cis*-dichloroethylene (*cis*-DCE), vinyl chloride (VC), and then to ethylene. VC biodegradation is the RD’s limiting step. Indeed, uncompleted anaerobic dechlorination might occur, resulting in VC accumulation, which is more toxic than the parent compounds (Dolinová et al., 2017). However, VC can be removed further through oxidative processes when specialized microorganisms are present in the aquifer.

Several organohalide-respiring bacteria (OHRB) are known for their dechlorinating ability. Among them, *Dehalococcoides mccartyi* is responsible for dechlorination of highly chlorinated solvents (i.e., PCE) to ethylene (Adrian et al., 2000; Maphosa et al., 2010; Hug et al., 2013; Jugder et al., 2016; Matturro et al., 2016). RD occurs through the metabolic activity of reductive dehalogenase enzymes (i.e., TceA, BvcA, and VcrA) typical of *D. mccartyi* strains involved in various RD steps (Saiyari et al., 2018). *D. mccartyi* (by 16S rRNA gene quantification) and reductive dehalogenase genes are commonly used as biomarkers for monitoring of RD processes, both on laboratory and field scales (Jugder et al., 2015; Fincker and Spormann, 2017). Moreover, several aerobic VC-assimilating bacteria have been isolated from CAH-contaminated groundwater (Fullerton et al., 2014; Liang et al., 2017). Some microorganisms can adapt to low oxygen concentrations and form aerobic niches in polluted aquifers (Coleman et al., 2002). Among them, ethylene-oxidizing bacteria (ethenotrophs) may utilize VC directly as a carbon and energy source; furthermore, they can co-metabolize VC while utilizing ethylene as a growth substrate (Verce et al., 2001; Jin and Mattes, 2008; Mattes et al., 2010). Moreover, methane-oxidizing bacteria (methanotrophs) fortuitously oxidize VC during aerobic growth in methane (Chang and Alvarez-Cohen, 1995; Semrau, 2011). The enzymes involved in VC oxidation are alkene monooxygenase (AkMO), whose alpha subunit is encoded by the *etnC* gene (Hartmans and De Bont, 1992; Verce et al., 2000), and epoxyalkane coenzyme M transferase (EaCoMT) encoded by the *etnE* gene (Coleman and Spain, 2003; Mattes et al., 2005). The *etnC* and *etnE* genes are currently used as biomarkers for monitoring ethenotroph populations on laboratory and field scales (Mattes et al., 2015). Thus, coupling reductive and oxidative dechlorination with engineered systems is a promising strategy for CAH removal, mainly because it can ensure the complete removal of highly chlorinated ethylenes (i.e., PCE), avoiding secondary contamination by more toxic and mobile low-chlorinated by-products (i.e., VC).

Among the bioremediation strategies proposed for restoration of groundwater contaminated by chlorinated solvents, bioelectrochemical systems (BESs) emerged in the past decade as an effective solution. BESs allow prompting reductive dechlorination, overcoming the use of fermentable organic substrates that can introduce uncontrollable side reactions (Wang et al., 2020). In BESs, electrons are directly provided (in the cathode) or accepted (in the anode) to perform the dechlorination process. Despite bioelectroremediation technologies having been deeply investigated and optimized under various conditions, little is still known about the composition and dynamics of microorganisms involved in reactions occurring in electrodes (Aulenta et al., 2008, 2010; Strycharz et al., 2010; Wang et al., 2015; Chen et al., 2018).

Notably, our research group has recently proposed an innovative BES aiming at sequential reductive/oxidative PCE dechlorination occurring in two separate reactors where PCE is first reduced (cathodic chamber) to TCE, *cis*-DCE, and VC, and then chlorinated by-products (i.e., VC) are oxidized (anodic chamber) to achieve complete PCE removal (Zeppilli et al., 2019, 2021; Dell’Armi et al., 2021a). The system has been tested with various PCE-contaminated feeding solutions, including an anaerobic mineral medium (MM) as the optimum condition on laboratory scale, synthetic groundwater (SG) to mimic the anion species content of contaminated aquifers, and, ultimately, real contaminated groundwater (RG) collected from an industrial site located in Northern Italy.

Here, we report the microbiome compositions and dynamics occurring in the sequential reductive/oxidative BES operated under various feeding conditions. Moreover, the main biomarkers of reductive (*D. mccartyi* 16S rRNA and *tceA*, *bvcA*, *vcrA* genes) and oxidative (*etnE* and *etnC* genes) dechlorination have been monitored and quantified. Possible metabolic mechanisms occurring in the BES have been discussed.

## Materials and Methods

### Reactor Configuration and Operating Conditions

The anaerobic/aerobic sequential BES analyzed in this study includes two tubular microbial electrolysis cells (MECs) used as reductive and oxidative reactors (Supplementary Figure 1). Both reactors adopted a membrane-free configuration. Each MEC was equipped with an internal graphite counter-electrode, with the aim to avoid electric shortcut and allow for electrolyte migration, and an Ag/AgCl electrode used as reference. The working electrode of the reductive reactor consisted of graphite granules, while in the oxidative reactor a commercial mixed metal oxide (MMO) electrode was employed as the working electrode (Magneto Special Anodes, Schiedam, Netherlands). The polarization of the working electrode of each reactor was controlled employing the Amel 459 and Biologic VSP 300 Potentiostat.

In this study, the following feeding solutions employed for the reactors’ operations were tested:

The mineral medium (MM), first adopted as feeding solution, was composed of 1 g/L NaCl, 0.5 g/L MgCl_2_⋅6H_2_O, 0.2 g/L KH_2_PO_4_, 0.3 g/L NH_4_Cl, 0.3 g/L KCl, 0.015 g/L CaCl_2_⋅2H_2_O, 0.05 g/L Na_2_S, 2.5 g/L NaHCO_3_, 1 ml/L metal solution, and 10 ml/L vitamin solution. The MM feeding solution was contaminated with PCE to obtain a theoretical concentration of 100 μM. During MM feeding, the cathodic potential for the reductive reactor was –550 mV vs. SHE, and the anodic potential for the oxidative reactor was +1.4 mV vs. SHE. The average flow rate for the system was 1.62 ± 0.06 L/d that resulted in HRT of 5.1 days.

The synthetic groundwater (SG) feeding solution was constituted by tap water added with sulfate and nitrate based on a previous Rho-contaminated groundwater characterization (200 mgSO_4_^2–^/L and 30 mgNO_3_^–^/L). The SG feeding solution for the reductive reactor was contaminated with PCE to obtain a theoretical concentration of 100 μM. Because during operation with the SG feeding solution the reductive and oxidative reactors worked separately, the SG for oxidative reactor feeding was contaminated with a theoretical *cis*-DCE concentration of 20 μM (Zeppilli et al., 2021). During SG feeding the working electrode potentials were –450 mV vs. SHE and +1.4 mV vs. SHE for the reductive and oxidative reactors, respectively. The average flow rate was 2.2 ± 0.7 L/d that led to a 3.7-day HRT.

Real groundwater (RG) was collected from a contaminated site located in northern Italy. The composition included. 233 mg/L total carbon, 186 mg/L inorganic carbon, 47 mg/L total organic carbon, 130 mg/L volatile fatty acids, 59 mg/L SO_4_^2–^, 31 mg/L Fe total, 20 mg/L Fe^3+^, 35 μg/L PCE, 47 μg/L TCE, 129 μg/L *cis*-DCE, and 43 μg/L VC. During RG feeding, the working electrode of the reductive reactor had a potential of –450 mV vs. SHE. The oxidative reactor was operated in galvanostatic mode with a fixed current between the working electrode and the counter electrode of +15 mA, which was similar to the flowing current of previous experimental conditions. The reactor was fed with an average flow rate of 4.6 ± 0.5 L/d, which corresponded to a hydraulic retention time (HRT) of 1.8 days.

### Analytical Methods and Calculations

Perchloroethylene, TCE, *cis*-DCE, and VC, together with methane and ethylene, have been quantified using a Dani Master gas chromatograph equipped with a flame ionization detector (FID). Determination of hydrogen, oxygen, and CO_2_ was performed with a Dani Master gas chromatograph equipped with a thermal conductivity detector (TCD). Anion concentrations, i.e., sulfate (SO_4_^2–^) and nitrate (NO_3_^–^), were made with an ion cromatograph (Dionex ICS-1000 IC, Sunnyvale, CA, United States) equipped with a conductivity detector. Fe^3+^ was determined with a Nanocolor ^®^ colorimetric kit with a concentration range of 0.02–3 mg/L Fe and was then analyzed on a Shimadzu spectrophotometer with wavelength set at 540 nm. The specific working conditions have already been reported in previously published studies. The most relevant process parameters have been calculated using the equations shown in Tables 1, 2 and already reported elsewhere. The Q represents the flow rate for the sequential system, F is the Faraday constant (96485 C/e- mol) and 86,400 are the seconds contained in 24 h. All the calculations were carried out considering the concentrations of each compound.

**TABLE 1:** Calculations for estimation of rates of the main processes occurring in the reductive reactor.

Parameters	Reductive reactor
RD rate (μeq/Ld)	$\left(\frac{Q}{V_{r\,e\,d\,u\,c\,t\,i\,v\,e\,r\,e\,a\,c\,t\,o\,r}}\right)*$
	([*TCE*]*2 + [cDCE]*4 + [VC]*6 + [ETH]*8)
Methanogenesis (meq/Ld)	$\left(\frac{Q}{V_{r\,e\,d\,u\,c\,t\,i\,v\,e\,r\,e\,a\,c\,t\,o\,r}}\right)*\left[CH_{4}\right]*$ 8
Sulfate reduction (meq/Ld)	$\left(\frac{Q}{V_{r\,e\,d\,u\,c\,t\,i\,v\,e\,r\,e\,a\,c\,t\,o\,r}}\right)*{\left[S\,O_{4}^{2-}\right]}_{R\,e\,m\,o\,v\,e\,d}*8$
Nitrate reduction (meq/Ld)	$\left(\frac{Q}{V_{r\,e\,d\,u\,c\,t\,i\,v\,e\,r\,e\,a\,c\,t\,o\,r}}\right)*{\left[N\,O_{3}^{-}\right]}_{R\,e\,m\,o\,v\,e\,d}*5$
Iron (III) reduction (μeq/Ld)	$\left(\frac{Q}{V_{r\,e\,d\,u\,c\,t\,i\,v\,e\,r\,e\,a\,c\,t\,o\,r}}\right)*{\left[F\,e^{3+}\right]}_{R\,e\,m\,o\,v\,e\,d}*1$

**TABLE 2:** Calculations for the estimation of efficiency in VC and *cis*-DCE removal occurring in the oxidative reactor.

Parameters	Oxidative reactor
VC removal efficiency (%)	$\frac{{\mathrm{VC}}_{\mathrm{in}}-{\mathrm{VC}}_{\mathrm{out}}}{{\mathrm{VC}}_{\mathrm{in}}}*100$
*cis*-DCE removal Efficiency (%)	$\frac{c\,D\,C\,E_{i\,n}-c\,D\,C\,E_{o\,u\,t}}{c\,D\,C\,E_{i\,n}}*100$

### Biomolecular Analysis: Sampling and DNA Extraction

Droplet Digital PCR (ddPCR) and 16S rRNA Gene Amplicon sequencing were performed on the DNA extracted from liquid samples collected from the outlet of the reductive and oxidative reactors under MM, SG, and RG feeding conditions. The samples were collected after 70 days of each feeding condition. In detail, 30 ml of effluent were collected from the outlet of each reactor and immediately filtered on polycarbonate filters (pore size 0.22 μm, 47 mm diameter, Nuclepore) to harvest the biomass. DNA was directly extracted from the filters using the DNeasy PowerSoil Pro Kit (Qiagen, Italy) following the manufacturer’s instructions. Purified DNA from each sample was eluted in 70 μl sterile Milli-Q and stored at –20°C for further biomolecular analysis.

### 16S rRNA Gene Amplicon Sequencing

The extracted DNA was employed for 16S rRNA gene amplicon sequencing, targeting the V1–V3 regions of bacterial 16S rRNA (Matturro et al., 2017). PCR reactions were performed in duplicate, in 25 μl total volume and including 4 ng of DNA template, 0.5 μM of library adaptors with V1–3 primers (27F: 5′-AGAGTTTGATCCTGGCTCAG-3′; 534R: 5′-ATTACCGCGGCTGCTGG-3′), and 1 × Phusion Flash High Fidelity Master Mix (Thermo Fisher Scientific, Waltham, MA, United States). The libraries were purified using an Agencourt AMpure XP bead according to protocol instructions (Beckman Coulter, United States). Library concentration was determined with the Qubit 3.0 Fluorometer (Thermo Fisher Scientific, Waltham, MA, United States). The purified libraries were pooled in equimolar concentrations and diluted to 4 nM. In order to overcome low complexity matter, 20% Phix control was added to the libraries. The samples were paired-end sequenced (2 × 301 bp) on a MiSeq platform (Illumina, San Diego, CA, United States) using a MiSeq Reagent kit v3 (Illumina), at 600 cycles, following the standard procedures for preparing and loading samples.

The quality of reads was verified with fastQC and, sequencing data were processed and analyzed using the QIIME2 software tools 2018.2 release (Caporaso et al., 2010). The reads were demultiplexed with demux plugin1, and deionized, dereplicated, and filtered from chimera with DADA2 algorithm (Callahan et al., 2016). Taxonomic analysis was based on a pre-trained naïve Bayes classifier based on the 16S rRNA gene database at a 99% similarity of the SILVA132 release (Quast et al., 2013). Raw data are deposited under BioProject PRJNA839891 with BioSample accession numbers SAMN28557297–SAMN28557302. The data are reported as relative percentage of single amplicon sequence variants (ASVs) out of the total reads. Shannon Index was calculated with PAST version 4.0.

### Quantification of Functional Genes by Droplet Digital ^®^ (ddPCR)

*Dehalococcoides mccartyi* 16S rRNA, the reductive dehalogenase genes (*tceA*, *bvcA*, and *vcrA*), and the e*tnC* and *etnE* genes were quantified by ddPCR. Absolute quantification assays were performed with the QX200™ Droplet Digital™ PCR System (Bio-rad, United States). The quantification assays included the preparation of a PCR reaction mixture for each targeted gene, which was then used to generate droplets with the QX200 Droplet Generator (Bio-Rad, United States). PCR amplification was performed on the droplets’ mixture with a T100 thermal cycler (Bio-Rad, United States), and quantitative data were read with QX200 Droplet Reader (Bio-Rad, United States).

Each PCR reaction mixture (22 μl total volume) included ddPCR Supermix for Probes ^®^ or ddPCR EvaGreen Supermix ^®^ (Bio-Rad, United States), 3 μl of DNA as a template, and 900 nM of each primer. In addition, 300 nM of TaqMan probe was added when required (i.e., *D. mccartyi* 16S rRNA; *tceA*, *bvcA*, and *vcrA* genes). The primers and probes used are listed in Table 3.

**TABLE 3:** Primers and probes used for ddPCR gene quantifications.

Target gene	Primers and probes	Sequence	Assay	References
*Dehalococcoides mccartyi 16S rRNA*	Dhc 1200F	5′-CTGGAGCTAATCCCCAAAGCT-3′	Supermix for Probes ^®^	He et al., 2003
	Dhc 1271R	5′-CAACTTCATGCAGGCGGG-3′		
	Dhc 1240-Probe	5′-FAM-TCCTCAGTTCGGATTGCAGGCTGAA-TAMRA 3′		
*tceA*	tceA 1270F	5′-ATCCAGATTATGACCCTGGTGAA-3′	Supermix for Probes ^®^	Johnson et al., 2005
	tceA 1336R	5′-GCGGCATATATTAGGGCATCTT-3′		
	tceA 1294-Probe	5′-FAM-TGGGCTATGGCGACCGCAGG-TAMRA 3′		
*vcrA*	vcrA 1022F	5′-CGGGCGGATGCACTATTTT-3′	Supermix for Probes ^®^	Ritalahti et al., 2006
	vcrA 1093R	5′-GAATAGTCCGTGCCCTTCCTC-3′		
	vcrA 1042-Probe	5′-FAM-CGCAGTAACTCAACCATTTCCTGGTAGTGG-TAMRA 3′		
*bvcA*	bvcA 925F	5′-AAAAGCACTTGGCTATCAAGGAC-3′	Supermix for Probes ^®^	Ritalahti et al., 2006
	bvcA 1017R	5′-CCAAAAGCACCACCAGGTC-3′		
	bvcA 977-Probe	5′-FAM-TGGTGGCGACGTGGCTATGTGG-TAMRA 3′		
*etnC*	etnC-F	5′-ACCCTGGTCGGTGTKSTYTC-3′	EvaGreen Supermix ^®^	Jin and Mattes, 2010
	etnC-R	5′-TCATGTAMGAGCCGACGAAGTC-3′		
*etnE*	etnE-F	5′CAGAAYGGCTGYGACATYATCCA-3′	EvaGreen Supermix ^®^	Jin and Mattes, 2010
	ernE-R	5′-CSGGYGTRCCCGAGTAGTTWCC-3′		

Twenty microliters of the PCR reaction mixtures was mixed with 70 μl of Droplet Generation Oil (Bio-Rad, United States) to generate droplets with an eight-channel DG8 cartridge. In a survey, 40 μl of the prepared droplets were then used for the PCR amplification in the T100 thermal cycler (Bio-Rad, United States) with the following cycling conditions: 10 min at 95°C, 39 cycles for 30 s at 94°C and 1 min at 60°C (ramping rate set to 2°C/s), 10 min at 98°C, ending at 4°C, for the *D. mccartyi* 16S rRNA, *tceA*, *bvcA*, and *vcrA* genes. The PCR cycling conditions for the *etnE* and *etnC* genes were 5 min at 95°C, 39 cycles for 30 s at 95°C and 1 min at 58°C (ramping rate set to 2°C/s), 5 min at 4°C, 5 min at 90°C, ending at 4°C. After the PCR amplification, quantification data were analyzed with the QX200 Droplet Reader (Bio-Rad, United States) to determine the positive and negative fluorescent droplets and calculate the targeted gene concentrations. The positive and negative droplets were divided by applying a fluorescence amplitude threshold. The data were analyzed using the QuantaSoft software (Bio-Rad, United States) by calculating the ratio of the positive droplets over the total droplets in each sample. The quantitative data were reported as gene copy numbers per volume of liquid sample (95% confidence intervals).

## Results

### Kinetic Performance of the Bioelectrochemical Reactors

In the reductive compartment, high PCE removal efficiencies corresponded to 97 ± 1% (MM), 95 ± 6% (SG), and 97 ± 2% (RG) (Table 4). The RD rate with MM feeding was equal to 99 ± 5 μeq/Ld, while the introduction of SG caused a decrement of the RD rate up to 68 ± 1 μeq/Ld. Moreover, the utilization of RG in the reactor yielded a low RD rate (0.51 ± 0.04 μeq/Ld) mostly due to the complexity of RG in terms of chemical composition and to the very low chlorinated ethylene concentrations in groundwater typical of an aged contaminated site (0.2 μM PCE, 0.34 μM TCE, 1.3 μM *cis*-DCE, 0.69 μM VC). Additionally, the reductive reactor effluent composition mostly depended on the operating conditions adopted in terms of applied potential, hydraulic retention time, and feeding solution composition. Indeed, during the feeding with MM, the effluent was composed by VC and ethylene, with average concentrations of 41 ± 7 and 19 ± 4 μmol/L (Zeppilli et al., 2021), while the shift to the SG promoted the change of the reductive reactor effluent, which resulted mostly composed by *cis*-DCE with an average concentration of 35 ± 4 and 4 ± 1 μmol/L (Dell’Armi et al., 2021b). In the oxidative reactor, the removal efficiency for medium and low chlorinated CAHs (i.e., *cis*-DCE and VC) was higher than 90% (Table 5).

**TABLE 4:** Reductive reactor performances with the three feeding solutions: Mineral Medium (MM), Synthetic groundwater (SG), and Real Groundwater (RG).

Reductive reactor	−550 mV vs. SHE	−450 mV vs. SHE	
Feeding solution	MM	SG	RG
RD Rate (μeq/Ld)	99 ± 5	68 ± 1	0.51 ± 0.04
PCE removal efficiency (%)	97 ± 1	95 ± 6	97 ± 2
Methanogenesis (meq/Ld)	0.34 ± 0.04	0.75 ± 0.19	–
Sulfate reduction (meq/Ld)	–	3.2 ± 0.5	0.25 ± 0.04
Nitrate reduction (meq/Ld)	–	0.3 ± 0.1	–
Iron (III) reduction (μeq/Ld)	–	–	945 ± 42

**TABLE 5:** Oxidative reactor performances with the three feeding solutions: Mineral Medium (MM), Synthetic groundwater (SG), and Real Groundwater (RG).

Oxidative reactor	+1.4 V vs. SHE		+15 mA
Feeding solution	MM	SG	RG
VC removal (%)	92 ± 2	–	92 ± 2
*cis*-DCE removal (%)	–	68 ± 5	100 ± 6

In addition to the dechlorination process, under the feeding with MM, no sulfate, nitrate, or iron reduction was observed (Table 4). Diversely, the use of SG stimulated sulfate reduction (3.2 ± 0.5 meq/Ld) and, to a lower extent, nitrate reduction (0.3 ± 0.1 meq/Ld) accordingly with the presence of sulfate and nitrate in SG. Methanogenesis was also observed under both MM (0.34 ± 0.04 meq/Ld) and SG (0.75 ± 0.04 meq/Ld) feeding. During the feeding with contaminated RG, only sulfate reduction (0.25 ± 0.04 meq/Ld) and significant iron reduction (945 ± 42 meq/Ld) were observed (Table 4).

### Microbiome Composition of the Cathodic Compartment

**FIGURE 1:**
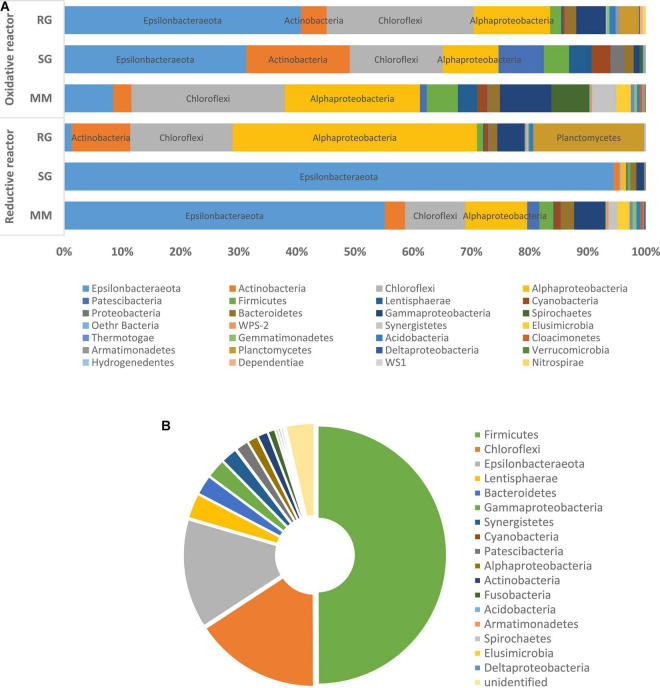
Microbiome composition at high phylogenetic levels (i.e., phylum or class) of the reductive and oxidative reactors under different feeding conditions. (A) Mineral medium (MM), synthetic groundwater (SG), and real groundwater (RG). (B) Microbiome composition at the phylum level of the RG collected from the contaminated site and used as feed for the BES.

**FIGURE 2:**
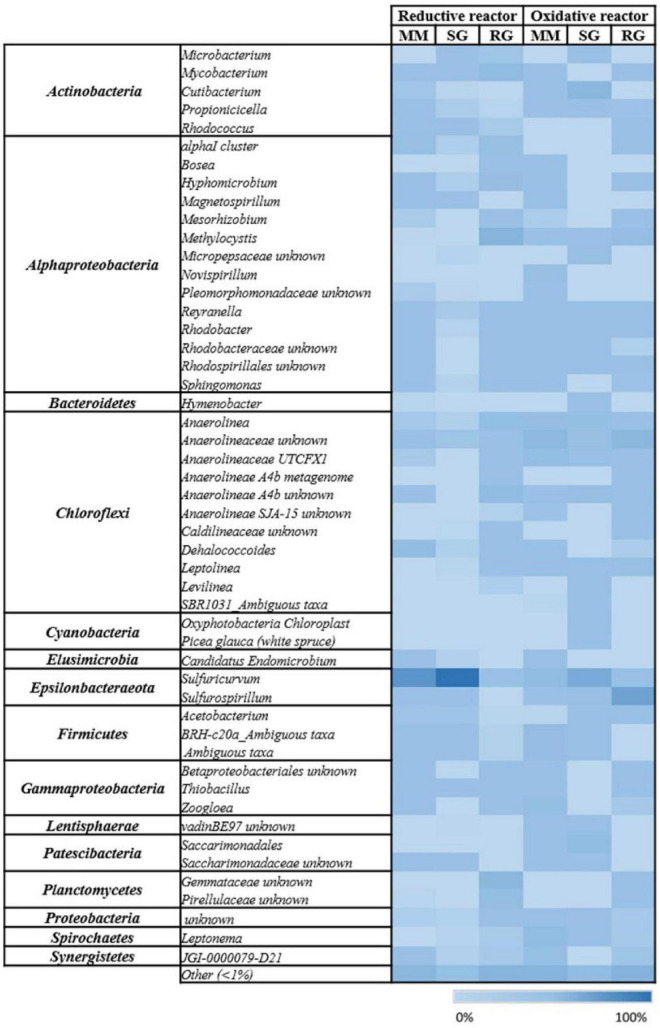
Heat map of the microbial communities at the genus level of the reductive and oxidative reactors under different feeding conditions: Mineral medium (MM), synthetic groundwater (SG), and real groundwater (RG). The color intensity in each cell shows the relative abundance of ASVs.

The 16S rRNA gene amplicon sequencing performed on the outlet of the cathodic compartment for the three feeding conditions tested revealed high diversity among the bacterial communities established (MM, H’ = 2.44; SG, H’ = 0.52; RG, H’ = 3.48) (Figure 1A). With the MM feeding, the microbial community showed the predominance of *Epsilonbacteraeota* (55.1%), mainly followed by *Alphaproteobacteria* (10.6%), *Cloroflexi* (10.3%), *Gammaproteobacteria* (5.2%), and to a minor extent, *Actinobacteria* (3.5%), *Firmicutes* (2.5%), and *Bacteroidetes* (2.3%) (Figure 1A). In more detail, majority of the ASVs detected in the *Epsilonbacteraeota* phylum were related to the facultative anaerobic *Sulfuricurvum* genus (54.6%). *Alphaproteobacteria* included members of the *Beijerinckiaceae alpha I cluster* (2.7%) known as facultative methylotrophs and methanotrophs, the denitrifying genus *Magnetospirillum* (1%), and members of the *Rhodobacteraceae* family (2.25%). *Cloroflexi* members were primarily associated to the *Dehalococcoides* genus (7.7%) and, to a lesser extent, the chemoorganotrophic *Anaerolineacae* family (2.2%) (Figure 2).

The shifting from MM to SG feeding, with the introduction of sulfate and nitrate into the reductive reactor, prompted a further increase in *Epsilonbacteraeota* that dominated the system (94.5%) and included members of the genera *Sulfuricurvum* (93.1%) and *Sulfurospirillum* (0.8%).

Diversely, the effluent of the reductive reactor fed with RG was composed of *Alphaproteobacteria* (42%), *Planctomycetes* (19%), *Chloroflexi* (17.6%), *Actinobacteria* (10%), *Gammaproteobacteria* (4.7%), *Bacteroidetes* (1.3%), and *Epsilonbacteraeota* (1.3%) (Figure 1A). In particular, *Alphaproteobacteria* were mainly represented by the genera *Methylocystis* (17%), *Hyphomicrobium* (4.2%), *Beijerinckiaceaealpha I cluster* (3.3%), *Reyranella* (2.2%), and *Rhodobacter* (2%). The phylum *Planctomycetes* included sequences affiliated with uncultured *Gemmataceae* (12.8%) and uncultured *Pirellulaceae* (5.9%). *Chloroflexi* members were mainly affiliated with the *Anaerolineaceae* family (9.5%), followed by *A4b* members (5.5%), and to a minor extent, *Dehalococcoidaceae* (0.2%) (Figure 2). Notably, the real groundwater used was also analyzed before introduction into the BES to evaluate the initial microbiome composition of the groundwater collected from the contaminated site. The RG microbial community was mainly composed of the *Firmicutes* (49.9%), *Cloroflexi* (15.8%), and *Epsilonbactereota* phyla (13.7%) (Figure 1B). The most abundant groups in the aquifer were represented by several uncultured bacteria including members of the *Clostridiaceae* family in *Firmicutes* (45.5%), members of the *Anaerolineaceae* family among *Chloroflexi* (14.9%), and members of the genus *Sulfuricurvum* (12.1%).

### Microbiome Composition of the Oxidative Compartment

The microbiome composition of the oxidative reactor showed higher biodiversity than the reductive compartment, especially when fed with the mineral medium (MM, H’ = 3.82; SG, H’ = 2.96; RG, H’ = 2.97).

The microbial community at the end of the operation with the MM was primarily composed of *Cloroflexi* (26%), *Alphaproteobacteria* (23%), *Gammaproteobacteria* (8.8%), *Epsilonbacteraeota* (8.4%), *Spirochaetes* (6.5%), and *Firmicutes* (5.3%) (Figure 1A). Moreover, the most abundant components were represented by members of the *Anaerolinaceae* family (23.5%), sulfur-oxidizing *Sulfuricurvum* (7.9%), denitrifiers *Leptonema* (6.4%) and *Zoogloea* (4.8%), *Novispirillum* (3.3%), and to a lower extent, members of *Rhodobacteraceae* (4.3%), *Methylocystis*, and *Mycobacterium* (1.3%) (Figure 2).

Due to change in the feeding solution, the SG bacterial community exhibited an increment in *Epsilonbacteraeota* (31%), primarily affiliated with the *Sulfuricurvum* genus (28%), and *Sulfurospirillum* (2.8%), followed by *Actinobacteria* (17.8%), *Cloroflexi* (16%), *Alphaproteobacteria* (9.5%), and the *Patescibacteria* phylum (7.8%) (Figure 1A). The *Actinobacteria* population was mainly composed of the *Propionibacteraceae* family (13.5%), while *Anaerolineaceae* members (13.2%) dominated the *Cloroflexi* phylum. Two uncultured *Saccharimonadales members* were also found as part of *Patescibacteria*. Similarly, even in the presence of RG, the composition of the microbiome included the *Epsilonbacteraeota* (40.7%), *Cloroflexi* (25.3%), *Alphaproteobacteria* (13.1%), *Gammaproteobacteria* (5%), and *Actinobacteria* (4.4%) phyla.

However, in this condition, the genus *Sulfurospirillum* (38.5%) enriched the anodic chamber, together with *Anaerolinaceae* members (17.4%), methanotroph *Methylocystis* (5.1%), ethenotroph *Mycobacterium* (1.4%), and sulfur-oxidizing *Thiobacillus* (1.4%) (Figure 2).

### Biomarkers for Reductive and Oxidative Dechlorination

**FIGURE 3:**
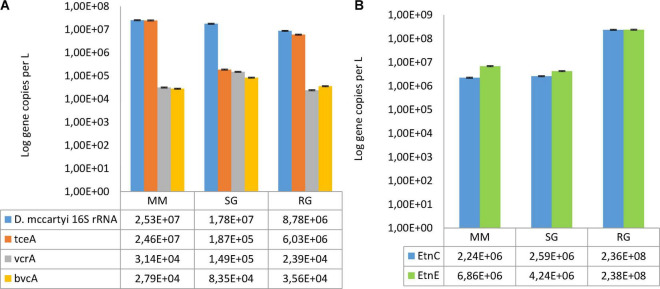
(A) Abundances of *Dehalococcoides mccartyi* 16S rRNA and the reductive dehalogenase genes (tceA, bvcA, and vcrA) estimated at the outlet of the reductive reactor. (B) Abundances of the functional genes (etnC and etnE) involved in VC oxidation estimated from the outlet of the oxidative reactor. Feeding conditions: Mineral medium (MM), synthetic groundwater (SG), and real groundwater (RG).

*Dehalococcoides mccartyi* was observed in the reductive rector under all the conditions explored (Figure 3A). Initially, by MM feeding, *D. mccartyi* 16S rRNA accounted for 2.35E + 07 gene copies/L, and a similar abundance was observed during the operation with SG (1.78E + 07 gene copies/L) but decreased by one order of magnitude under RG feeding (8.78E + 06 gene copies/L). Moreover, *tceA* was the most abundant reductive dehalogenase gene found in all the conditions explored, while the *vcrA* and *bvcA* genes were less abundant. In detail, *vcrA* ranged from 2.39E + 04 gene copies/L in RG to 1.49E + 05 gene copies/L in SG, whereas *bvcA* concentration showed limited variations among the three conditions (from 2.79E + 04 to 8.35 E + 04 gene copies/L). Furthermore, the functional genes associated to VC degradation (*etnC* and *etnE*) were detected in the oxidative reactor effluent (Figure 3B). The abundance of *etnC* ranged from 2.24E + 06 gene copies/L (with MM) to 2.36E + 08 gene copies/L (with RG). Similarly, the quantification of the *etnE* gene revealed a two-order-of-magnitude increment under the RG feeding (2.38E + 08 gene copies/L) when compared with the previous conditions (6.86E + 06 and 4.24E + 06 gene copies/L with MM and SG, respectively).

## Discussion

In this study, the microbiome established in a BES for sequential anaerobic/aerobic PCE dechlorination was characterized under various feeding conditions and at increasing level of complexity in terms of anion content and possible effects on redox reactions. Together with microbial characterization of the effluent of the reductive and oxidative compartments, the abundances of the biomarker genes involved in reductive (*D. mccartyi* and reductive dehalogenase genes) and oxidative (*etnE* and *etnC*) dechlorination were also estimated under all the tested conditions.

The various feeds affected the reactor performances and microbiome compositions.

The use of the MM feed showed the best kinetic performances as expected having the optimum composition for cultivation of anaerobic dechlorinating cultures. Indeed, the MM feed was also used for start-up of the reactor and acclimation of the bacterial community in the BES (Zeppilli et al., 2019, 2021), ensuring high PCE removal efficiencies and avoiding the occurrence of competitive reactions such as sulfate or nitrate reduction. Diversely, the use of SG, where sulfate and nitrate were added, caused a slight decrement in PCE removal efficiency introducing sulfate and nitrate reduction processes. Nevertheless, as highlighted in a previous study, the presence of sulfate and nitrate reduction reactions in the cathodic compartment of the BES did not involve a competition for the reducing power with the RD reaction (Dell’Armi et al., 2021a). In line with kinetic performances, in the reductive compartment of the BES fed with MM or SG, *D. mccartyi* was abundant (2.5E + 07 and 1.8E + 07 16S rRNA gene copies/L respectively under MM and SG feeding) and was mainly represented by *tceA*-carrying strains. *D. mccartyi* was responsible for the biological RD occurring in the reductive compartment, likely *via* an indirect electron transfer mechanism, but this microorganism has not been considered an electroactive bacterium until now (Matturro et al., 2021). Regarding microbiome composition, *Epsilonbacteraeota* members were dominant (50.8 and 89.5% under MM and SG, respectively) and mostly affiliated to one ASV that showed 99.5% of identity to *Sulfuricurvum kujiensestrain YK-3* (GenBank: AB080644) (Kodama and Watanabe, 2003). The *Sulfuricurvum* species is frequently found in hydrocarbon-contaminated environments, and it is a facultative anaerobic, chemolithoautotrophic, sulfur-oxidizing, and nitrate-reducing bacterium (Kodama and Watanabe, 2004; Han et al., 2012). It can be grown anaerobically using sulfide, elemental sulfur, thiosulfate, and hydrogen as electron donors and nitrate as an electron acceptor under anaerobic conditions (Kodama and Watanabe, 2004). The system configuration might explain the role of the *Sulfuricurvum* species found in the reactor. Indeed, as mentioned in previous studies, the unrecovered current of the system is likely associated with parasitic reductive/oxidative reactions occurring between the working electrode (cathode) and the inner counter electrode (anode) that promotes electron loops (Zeppilli et al., 2019). Therefore, some ionic species can be exchanged between different parts of the reactor, establishing simultaneous reductive and oxidative reactions (Zeppilli et al., 2021). Thus, hydrogen sulfide, already present in the MM feeding solution or obtained by sulfate reduction occurring during SG feeding, could be bioelectrochemically oxidized into elemental sulfur and then oxidized by the *Sulfuricurvum* species by nitrate reduction (Supplementary Figure 2). However, in order to confirm the role of the *Sulfuricurvum* species found in the BES and its metabolic functions and interactions, additional whole metagenomic analyses are necessary. Moreover, the occurrence of methylotrophs and methanotrophs microorganisms under MM or SG feeding is in line with the methane production observed in the reactor. However, defining separately the anaerobic or aerobic metabolic pathways occurring in each chamber of the BES is challenging because of the presence of a counter electrode in each reactor of the BES that allow the establishment of various redox niches and, consequently, co-existence of oxidizing and reducing microorganisms in the chamber that are not separable. Indeed, sampling for biomolecular analysis was possible on the outlet of each reactor and obviously included all microorganisms present in the bulk.

The RG feeding caused a change in the microbiome composition of the reductive compartment involving higher biodiversity and decrement in *D. mccartyi* abundance (8.78E + 06 16S rRNA gene copies/L, decreased by one order of magnitude) compared to the previous feeding conditions. Organisms affiliated with *Clostridiaceae*, rich in the original community of the aquifer, decreased considerably their relative abundance in the reductive reactor at the end of the RG operation.

On the other hand, in the *Alphaproteobacteria* group, *Beijerinckiaceae* was the most abundant family with RG feeding, mainly followed by *Gemmataceae*, *Pirellulaceae*, and *Mycobacteriaceae*. The presence of low-chlorinated intermediates in RG may have stimulated the selection of this community in the cathodic chamber. The methanotrophic bacterial group of *Beijerinckiaceae* included the genera *Methylocystis*, *alphaI cluster*, and *Bosea*. Interestingly, the *Methylocystis* genus comprises aerobic, obligate, and facultative methylotrophic and methanotrophic bacteria capable of utilizing methane and methanol as sole carbon and energy sources (Jung et al., 2020). Members of this genus inhabit different soils, peatlands, and freshwater sediments, and they have been previously shown to be capable of degrading TCE and *cis*-DCE *via* aerobic metabolic or co-metabolic pathways (Uchiyama et al., 1989; Nakajima et al., 1992; Dolinová et al., 2017). *Pirellula*-like and *Gemmataceae* are chemoorganotrophic and ubiquitous aquatic bacteria are often detected in anoxic or micro-oxic habitats despite taxonomy describing representatives of these bacteria as strictly aerobic (Fuerst, 1995; Kulichevskaya et al., 2020). Conversely, diverse strains of *Mycobacterium* sp. have been previously associated with aerobic ethyleneotrophs considering their ability to grow on VC as the sole carbon and energy source (Mattes et al., 2010). These bacteria are indigenous and widely distributed in chlorinated-ethylene-contaminated sites adapted to low oxygen level (Coleman et al., 2002). Moreover, the presence of the *Rhodobacter* genus, known for its excellent iron reduction ability, may explain the iron reduction reaction, a characteristic of this condition (Weber et al., 2006).

Regarding the oxidative reactor, the MM community showed abundance of the *Leptospiraceae*, *Rhodobacteraceae*, and *Rhodocyclaceae* families. The presence of the *Rhodocyclaceae* family is of great interest, because its members are commonly found in polluted aquifers, which are involved in denitrification processes and in degradation of a wide range of aromatic compounds (Oren, 2014). In addition, the genera *Mycobacterium* and *Methylocystis* were present in this condition, in accordance with the high *cis*-DCE and VC removal efficiency achieved.

Conversely, the aerobic community with SG feeding included members of the *Propionibacteriaceae* family, uncultured *Saccharimonadales* and *Sulfurospirillum*, and the more abundant genus *Sulfuricurvum*. Members of the *Propionibacteriaceae* family have been isolated from a broad range of different habitats including chlorosolvent-contaminated soils and groundwater, but they deserve more investigations on their biodegradation potential (Bae et al., 2006; Stackebrandt, 2014). Likewise, very little is known about the ecology of the *Saccharimonadales* group despite *Saccharibacteria* having been shown to possess very small genomes and cell sizes, and it has therefore been proposed to live in symbiosis with other microorganisms depending on co-metabolism (Lemos et al., 2019). The *Sulfurospirillum* genus instead, consists of versatile, often microaerophilic bacteria growing with many different growth substrates. Few strains are able to use halogenated compounds as electron acceptors. The wide range of possible growth substrates enables many *Sulfurospirillum* species to thrive in polluted habitats, which is reflected by the presence of these bacteria in many contaminated sites (Goris and Diekert, 2016). This versatile metabolic capability suggests that it might be involved in the global dechlorination capability of the microbiome.

When the oxidative reactor was fed with RG, further enrichment of the *Sulfurospirillum* genus (38%), *Rhodocyclaceae* family members, *Mycobacterium*, and *Methylocystis* was obtained. All these microorganisms having good degradative capacities may have contributed to the removal of chlorinated compounds, in accordance with the excellent VC and *cis*-DCE removal efficiency detected under this feeding condition (Table 5). Besides, the presence and abundance of VC-oxidizing bacteria were further confirmed by the absolute quantification of functional genes involved in the oxidative dechlorination pathway, which increased by two orders of magnitude (up to 2.38E + 08 gene copies/L) in this feeding condition compared to the previous ones.

Overall, our findings are in line with various studies conducted on BESs applied to contaminated groundwater that highlighted the presence of complex interactions in the sulfur cycle during the electrobioremediation process (Rakoczy et al., 2013; Daghio et al., 2016, 2017). In bioelectrochemical treatments, in fact, many substances may act as electron acceptors besides the anode, such as sulfate, nitrate, and iron(III) (Zhang et al., 2010). They capture electrons during the substrate degradation process and participate in the cycles of nitrogen, sulfur, and iron. For instance, sulfate may be reduced to sulfide, and sulfide can be oxidized to elemental sulfur through biological or chemical processes. Sulfur may then be reduced again to sulfide, which can be re-oxidized, thus constituting a sulfur cycle that promotes current generation as reported in the literature (Dutta et al., 2009). Thus, sulfate/sulfide can serve as an electron shuttle to deliver electrons from microorganisms to the electrode and vice versa (Zhao et al., 2006; Dutta et al., 2009; Rakoczy et al., 2013).

## Conclusion

This study highlighted that the feeding solutions affected the microbial composition of the BES and the rate of its dechlorination activities. The peculiarity of the BES configuration allowed for the simultaneous presence of diverse redox niches in each compartment allowing for complete PCE and by-product dechlorination. The complex interactions between these degrading microorganisms ensured excellent system performance, even with the RG. Despite sulfate, nitrate, and iron reduction reactions having not directly competed for reducing power with the RD process, the abundance of these anions promoted in the reductive reactor the predominance of some microbial groups linked with the sulfur cycle, especially members of the *Sulfuricurvum* genus. The microbiome in the oxidative compartment included both ethyleneotroph and methanotroph populations such as *Mycobacterium* and *Methylocystis.* They are able to achieve metabolic and co-metabolic degradation of low-chlorinated products, effectively preventing VC accumulation in the system. Overall, the sequential anaerobic/aerobic bioelectrochemical system fed with RG showed a great dechlorination potential, leading to the complete mineralization of chlorinated pollutants, with relevant implications for system optimizations on a field scale for sustainable and cost-effective bioremediation.

## Data Availability Statement

The data presented in this study are deposited in the GenBank repository, accession number: PRJNA839891 (BioSample accession numbers: SAMN28557297–SAMN28557302).

## Author Contributions

MD, BM, MZ, and ED: investigation, validation, and writing of the original draft. SC: investigation and validation. MM, MP, and SR: supervision and funding acquisition. All authors contributed to the article and approved the submitted version.

## Conflict of Interest

The authors declare that the research was conducted in the absence of any commercial or financial relationships that could be construed as a potential conflict of interest.

## Publisher’s Note

All claims expressed in this article are solely those of the authors and do not necessarily represent those of their affiliated organizations, or those of the publisher, the editors and the reviewers. Any product that may be evaluated in this article, or claim that may be made by its manufacturer, is not guaranteed or endorsed by the publisher.
