# Supplementary material for: Microbiome Composition and Dynamics of a Reductive/Oxidative Bioelectrochemical System for Perchloroethylene Removal: Effect of the Feeding Composition
Source: Front Microbiol. 2022 Jul 18;13:951911. doi: 10.3389/fmicb.2022.951911 (PMC9340161; doi:10.3389/fmicb.2022.951911)
Supplement: Supplementary file 1 [file Data_Sheet_1.PDF]

Microbiome composition and dynamics of a reductive/oxidative bioelectrochemical system for PCE removal: effect of the feeding composition

M. L. Di Franca[1], B. Matturro[1]*, S. Crognale[1], M. Zeppilli[2], E. Dell'Armi[2], M. Majone[2], M. Petrangeli Papini[2], S. Rossetti[1]

[1]Water Research Institute (IRSA-CNR), Via Salaria km 29.300, 00015 Monterotondo RM, Italy
[2]Department of Chemistry, Sapienza University of Rome, P. le Aldo Moro 5, 00185 Rome, Italy
*** Correspondence:** bruna.matturro@irsa.cnr.it

Keywords: bioelectroremediation, chlorinated ethylenes, groundwater remediation, oxidative dechlorination, PCE, reductive dechlorination.

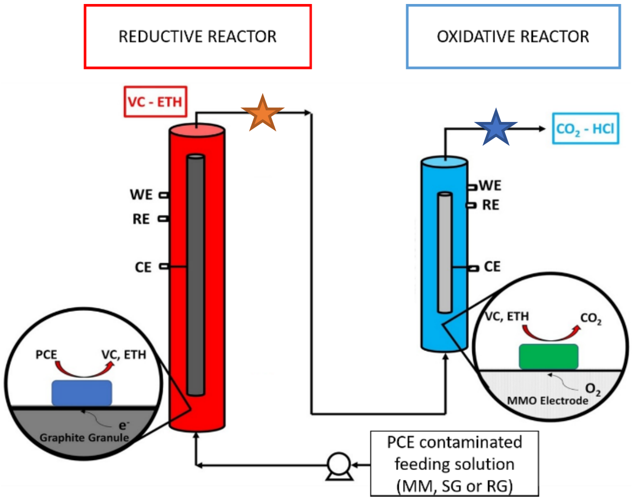

Figure S1. Configuration of the sequential reductive/oxidative BES for PCE removal. The BES has a counter electrode (CE), a working electrode (WE) and a reference electrode (RE) for each compartment. Stars represent the sampling points for the biomolecular analysis at the outlet of the reductive and oxidative reactors.

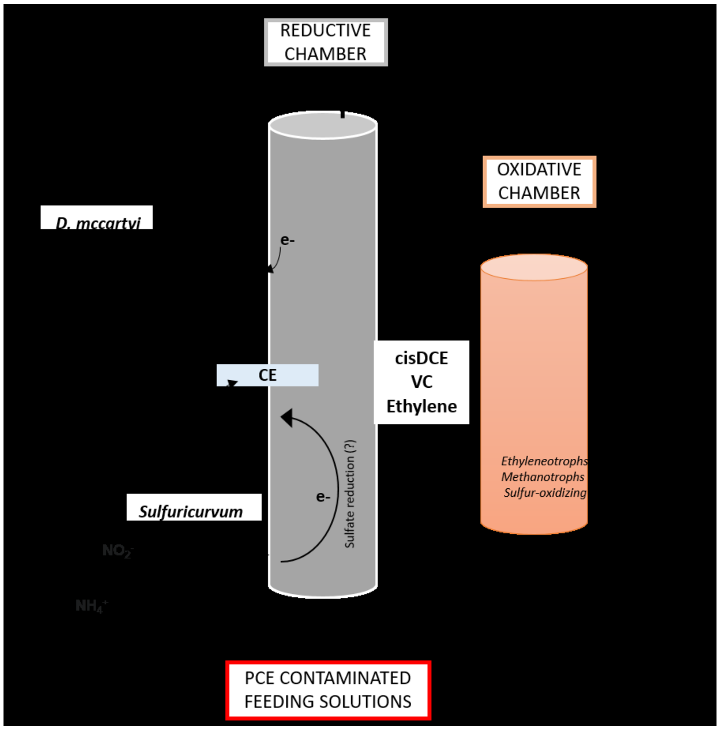

a.

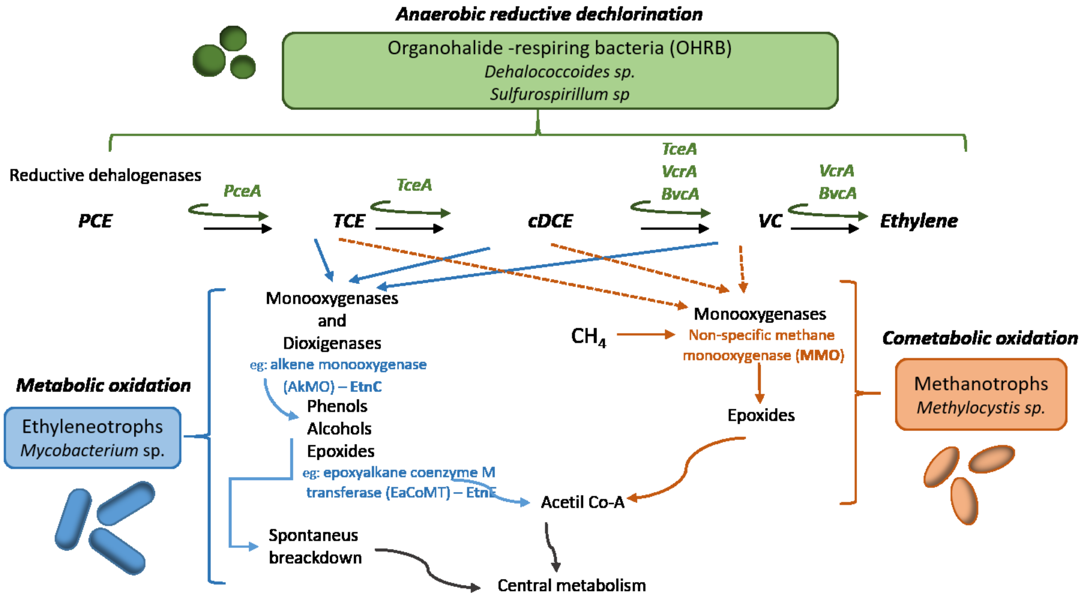

b.

Figure S2. Possible metabolic roles of the most abundant microorganisms under various feeding conditions (a) and functions within the main processes occurring in the BES (b).
